# Macrophages in Colorectal Cancer Liver Metastases

**DOI:** 10.3390/cancers11050633

**Published:** 2019-05-07

**Authors:** Nina Cortese, Cristiana Soldani, Barbara Franceschini, Marialuisa Barbagallo, Federica Marchesi, Guido Torzilli, Matteo Donadon

**Affiliations:** 1 Department of Immunology and Inflammation, Humanitas Clinical and Research Center, 20089 Rozzano, Italy; nina.cortese@humanitasresearch.it (N.C.); marialuisa.barbagallo@humanitasresearch.it (M.B.); federica.marchesi@humanitasresearch.it (F.M.); 2 Department of Hepatobiliary and General Surgery, Humanitas Clinical and Research Center, 20089 Rozzano, Italy; cristiana.soldani@humanitas.it (C.S.); barbara.franceschini@humanitas.it (B.F.); guido.torzilli@hunimed.eu (G.T.); 3 Department of Biotechnology and Translational Medicine, University of Milan, 20090 Segrate, Italy; 4 Department of Biomedical Science, Humanitas University, 20089 Rozzano, Italy

**Keywords:** macrophages, colorectal liver metastases, immune landscape

## Abstract

Tumor-associated macrophages (TAMs) provide a nurturing microenvironment for metastasis and are concomitantly key determinants of the efficacy of anticancer strategies. TAM represent an extremely heterogeneous population in terms of cell morphology, functions, and tissue localization. Colorectal liver metastases (CLM) display a high heterogeneity, responsible for a wide array of clinical presentations and responsiveness to treatments. In the era of precision medicine, there is a critical need of reliable prognostic markers to improve patient stratification, and, for their predominance in metastatic tissues, TAMs are emerging as promising candidates.

## 1. Introduction

Immune cells are essential components of the tumor microenvironment (TME) and a key determinant of metastasis [1,2,3,4,5]. A wealth of studies has revealed the association of specific immune signatures in the tumor microenvironment with post-operative tumor progression and metastasis [4,6,7,8] and tumor-associated macrophages (TAMs) [2,9,10]. Most of the work done until now has focused on primary tumors, while we have just started to unearth the complexity of microenvironment in metastases. By increasing our appreciation of the importance of immune profiles in dictating clinical outcome, these studies are slowly modifying our approach to cancer classification.

Among the variety of inflammatory cells populating the tumor microenvironment, this review will focus on TAMs. Besides contributing to the complexity of the TME, they have been shown to modify the efficacy of anticancer strategies, including targeted therapies, commonly used in the management of colorectal liver metastases (CLM) patients. For this reason, a clearer definition of their contributions could have relevant clinical implications in the management of cancer patients. Here, we will discuss the occurrence of TAMs in the microenvironment of human CLM, their ability to promote diverse immune responses, and their relevance as prognostic immune markers.

## 2. Macrophage Functions in the Tumor Microenvironment

Myeloid cells, including macrophages and other professional phagocytes, are essential elements of the TME, and their biology has been the object of numerous studies, both in preclinical models and in human cancer tissues. Among others, macrophages are the cells most frequently found within a tumor [5,11], with a variable impact on clinical outcome ranging from being pro-tumor [12] to being anti-tumor [10,13,14]. This duality is not surprising, considering that macrophages profoundly reprogram their functions in response to a wide variety of signals, both in physiological and in pathological conditions. An additional level of complexity is conferred by the tissue-specific peculiarities displayed by macrophages. In fact, macrophages are found in all tissues in the body, where they contribute to the maintenance of tissue homeostasis, performing a wide variety of functions [15]. A wealth of studies has shown the heterogeneity of tissue-resident macrophages, an important element that further increases the diversity of TAMs in tumors colonizing distinct tissues [15]. Thus, although macrophages in cancer derive from circulating monocytes recruited by the inflammatory, cancer-associated environment, they have to coexist with resident macrophages established prenatally. To what extent the different origins of these two components affect their function remains to be elucidated and could have major clinical implications. To this regard, single-cell based approaches could help to decipher the heterogeneity of TAM populations. 

### 2.1. Pro-Tumor and Anti-Tumor Roles

Despite the fact that macrophages, when properly activated by inflammatory stimuli, acquire the capacity to kill tumor cells [16,17], most frequently, the immunosuppressive microenvironment impedes the execution of this function. As a result, TAMs often emerge as pro-tumor players, promoting tumor growth and metastasis, sustaining angiogenesis and matrix remodeling, and secreting growth factors and immunosuppressive cytokines [18,19,20,21]. The paradoxical pro-tumor behavior of macrophages can be ascribed to the opposite actions that they have to fulfill in homeostatic conditions during the inflammatory response: in the first phase, they contribute to building up an inflammatory milieu that favors pathogen killing and elimination of the injurious stimulus, whereas in the second phase, they orchestrate the wound-healing response associated with the resolution phase of inflammation. This pro-healing attitude entails functions such as angiogenesis, matrix deposition, tissue remodeling, which, if prolonged and sustained, eventually evolve into tissue chronic inflammation. This scenario is exploited by tumor cells to promote tumor growth and metastasis.

When polarized towards a classically activated phenotype, macrophages are able to exert anti-tumor activities; this comprises an increase in direct killing of tumor cells, production of pro-inflammatory cytokines, and an increase of antigen presentation abilities. Moreover, monocytes expressing CD40 have been shown to upregulate major histocompatibility complex (MHC) class II, inducible nitric oxide synthase (iNOS), and tumor necrosis factor (TNF) after engagement of CD40 ligand (CD40L) on T cells [22].

On the other hand, the pro-tumor functions of macrophages can be simplistically classified as promoting tumor cell growth, mediating immune suppression, and facilitating the metastatic process. In the context of chronic inflammation, TAMs can create a mutagenic microenvironment that favors cancer initiation via the activation of transcription factors, including NFkB and STAT3, leading to the production of inflammatory products such as Interleukin 6 (IL-6), Interferon γ (IFNγ), TNF, growth factors, reactive oxygen/nitrogen species, and proteases [23]. Also, TAMs participate in the creation of a vascular network in growing tumors by secreting pro-angiogenic factors such as vascular endothelial growth factor A (VEGFA) [24] and chemokines (CXCL8, CXCL12) [25]. Furthermore, they help recruit and activate endothelial cells, fibroblasts, and pericytes through the production of transforming growth factor α (TGF-α) and TNF [26]. A subset of macrophages found adjacent to vessels and characterized by the expression of angiopoietin 1 receptor (TIE2) was identified as key mediator of tumor neo-angiogenesis; indeed, depletion of these cells inhibited tumor growth and metastasis [27].

Another tumor-promoting role of TAMs can be ascribed to their proclivity to create a suppressive immunologic microenvironment. In fact, macrophage polarization and function are strongly influenced by immunosuppressive signals and the predominant presence of Type 2 helper T (Th2) cells [5,28], resulting in the suppression of an effective anti-tumor response. 

This comprises various mechanisms that result in the negative modulation of natural killer (NK) cells and T cells. TAMs express inhibitory receptors, such as the non-classical MHC-I molecules HLA-E and HLA-G, and the immune checkpoint ligands programmed death-ligand 1 (PD-L1), PD-L2, B7-1 (CD80), and B7-2 (CD86) [5]. They also contribute to maintain an immunosuppressive environment by secreting IL-10 and TGFβ, which inhibit CD4 and CD8 T cells and induce regulatory T (Treg) cells [29]. Moreover, TAMs also recruit Treg cells through the production of numerous chemokines and dampen T cell cytotoxicity by depleting L-arginine via arginase 1 release or by depleting tryptophan via indoleamine 2,3-dioxygenase (IDO) expression [20].

The pro-metastatic functions of macrophages will be better discussed in the next paragraphs.

### 2.2. Macrophages in Anticancer Strategies

Several studies have shown that TAMs, beyond contributing to tumorigenesis, can alter the efficacy of standard anticancer therapies, such as chemotherapy and radiotherapy, as well as of unconventional targeted therapies [5,11,30,31]. Dichotomous behaviors for TAMs have been reported, both synergizing and hampering the efficacy of therapy, reflecting the complexity of macrophage functions. 

Several mechanisms have been suggested for TAMs synergizing with chemotherapy. Certain cytotoxic agents, including doxorubicin, oxaliplatin, and cyclophosphamide, induce the release from tumor cells of “eat-me” signals (for example, extracellular ATP, heat shock proteins, secreted type I interferon, extracellular nucleic acids, and many others still being unearthed) [32]. These signals stimulate phagocytes and antigen-presenting cells to drive anti-tumor responses by T cells, a process termed “immunogenic cell death” (ICD) [5,30]. In pancreatic [9] and colorectal cancer [10], a second mechanism involving the repolarization of macrophage phenotype by chemotherapy has been shown. On the other hand, macrophages have also been reported to hamper chemotherapy efficacy; this is often a direct consequence of typical macrophage functions, such as orchestrating an immunosuppressive response, and tissue-repair-related functions [33,34]. Radiotherapy strongly affects the tumor microenvironment, impacting on the overall type of immune response generated. Indeed, the innate immune system is stimulated by the release of inflammatory cytokines, namely, IL-1 and TNF, and pro-fibrotic factors, namely, TGF-β, which recruit monocytes and induce an immunosuppressive and tissue-repair phenotype in macrophages, thus contributing to tumor progression [5,23]. However, neoadjuvant low-dose irradiation was shown to curtail pro-tumoral macrophage functions such as immunosuppressive and proangiogenic activities [22]. Taken together, these studies evidence that macrophages have the potential to both synergize and hamper the efficacy of radiotherapy according to the context.

Immunomodulatory therapies have now entered clinical routine for an increasing number of cancers [35]. The most remarkable targets are the regulatory immunological checkpoint molecules cytotoxic T-lymphocyte-associated protein 4 (CTLA-4) and programmed cell death protein 1 (PD-1)/PD-L1; however, many more will soon be subjected to clinical assessment. The main mechanism of action of these checkpoint blockade antibodies depends on the counteraction of the negative signal induced in T cells upon binding of the corresponding ligand on tumor cells or antigen-presenting cells. In fact, macrophages express the ligands for PD-1 (B7H4, PD-L1, PD-L2) and for CTLA-4 (B7-1 and B7-2) and are, therefore, strongly involved in the immunosuppressive pathways targeted by checkpoint blockade inhibitors. The expression of triggers of checkpoint blockade, such as PD-L1, could be exploited as a tool to predict the response to immunotherapy. Moreover, mechanistic studies have shown that macrophages are also able to increase the activity of checkpoint inhibitors through molecules of the FcγR family. In particular, in melanoma, both in preclinical and in clinical settings, treatment with antibodies directed against CTLA-4 and glucocorticoid-induced TNFR-related protein (GITR) induced Treg depletion in an fragment crystallizable (Fc)-γ receptor (FcγR) -mediated manner [36,37].

Targeted therapies with monoclonal antibodies against a wide spectrum of tumor antigens represent a very promising tool in cancer therapy. Mechanistically, they can act in several ways, either directly by inducing apoptosis or inhibition of growth of tumor cells, or indirectly through the involvement of the immune system via complement activation or FcR-mediated innate responses. Classically, the immune effector function of monoclonal antibodies has been ascribed to NK cells because of their ability to carry out antibody-dependent cell-mediated cytotoxicity (ADCC). However, whereas NK cells mainly express FcγRIIIa, macrophages express all types of Fcγ receptors and are therefore also crucial effectors of therapeutic antibodies. In fact, growing evidence strongly suggests a role for macrophages in the elimination of tumor cells via antibody-dependent phagocytosis (ADP) [38]. Furthermore, macrophages reside in all tissues throughout the body, and their sheer abundance in the tumor microenvironment makes them singularly suitable as effectors of antibody-based therapies. Indeed, contributions to the efficacy of therapy by FcR-mediated immune response were reported for several antibodies, including Rituximab, a monoclonal antibody anti-CD20 used for lymphoma and leukemia treatment, Cetuximab, targeting the epidermal growth factor receptor (EGFR), Trastuzumab, an antibody directed against the epidermal growth factor receptor HER2/Erbb2, and Daratumumab, an anti-CD38 antibody for myeloma [39]. Along the same lines, functional polymorphisms in the human FcγRIIIA gene were found to correlate with response rates in patients with lymphoma, breast cancer, and metastatic colorectal cancer (CRC) treated with targeted therapies, strongly suggesting an impact of this polymorphism on ADCC by NK cells and myeloid cells [38].

Based on the evidence that macrophage-mediated phagocytosis critically contributes to the efficacy of many clinically approved therapeutic antibodies, currently, several approaches to increase macrophage contribution to response are under investigation. One notable example is represented by the recent studies on the CD47/SIRPα axis. Signal regulatory protein α (SIRPα) is considered a myeloid checkpoint molecule, as it is a negative regulator of macrophage phagocytosis; CD47 is expressed on many cell types and, upon engagement of SIRPα on macrophages, is perceived by phagocytes as a “don’t eat me signal”. Beyond its physiological role, CD47 is overexpressed by tumor cells, induced by oncogenic activation of *c-MYC*, as an escape strategy from macrophage phagocytosis and is currently under evaluation for therapeutic targeting, with promising results. The CD47/SIRPα axis has been targeted with anti-CD47 blocking antibodies and non-functional engineered SIRPα variants, in combination with anti-tumor therapeutic antibodies such as Rituximab, Cetuximab, and Trastuzumab, displaying synergistic activity in augmenting macrophage phagocytosis [40].

### 2.3. Clinical Relevance of Macrophages

**Figure 1:**
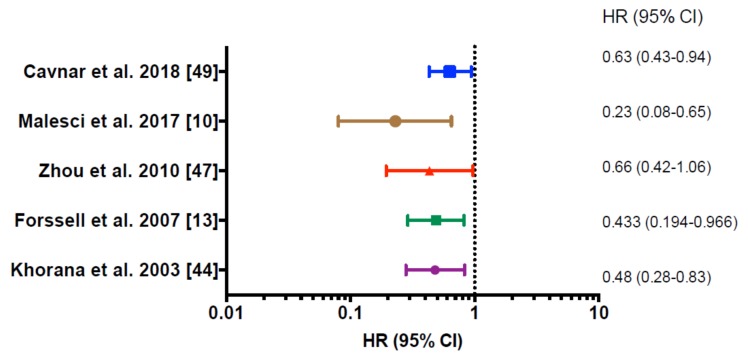
Forest plot showing significant prediction for survival in multivariate regression analysis of different clinical studies on colorectal cancer. HR, Hazard Ratio; 95% CI 95%, confidence interval. Hazard ratios <1.00 represent a decreased probability of death, hazard ratios >1.00 represent an increased probability of death; *p* < 0.05 was considered statistically significant.

Many studies have addressed the prognostic function of macrophages in human cancer. TAMs represent an extremely heterogeneous population in terms of cell morphology, functions, and localization within the tumor microenvironment. Studies investigating their prognostic significance have relied on a variety of methodological approaches, ranging from morphological identification in early efforts [17] to immunohistochemical analysis. The most commonly used marker to identify human macrophages is the pan-macrophage marker CD68. However, relying on CD68 for macrophage identification inevitably overlooks the prognostic potential of macrophage subsets with different functions in the tumor microenvironment. Moreover, it has been reported that stromal cells and cancer cells can also occasionally express CD68, therefore, this molecule should be carefully assessed [25]. More recent analyses have tried to infer the presence of macrophages across malignancies using gene expression profiles [41]. Consistent with the well-known role of macrophages in tumor promotion, several meta-analyses reported that a high density of TAMs correlated with poor overall survival in many human cancer types, including breast, bladder, prostate, head and neck, glioma, melanoma, and non-Hodgkin lymphoma [42]. However, this is not true for very few cancer types, such as colorectal (Figure 1) and gastric cancer, where high numbers of TAMs were found to associate with better prognosis [9,10,13] (Table 1). 

It should be acknowledged that these findings present with the limitation of not taking into account many factors (cancer subtype, anatomical location, developmental origin of macrophage subsets) that likely contribute to intratumoral and intertumoral diversity of TAMs. Collectively, many relevant studies have highlighted the heterogeneity of TAMs in the tumor microenvironment [43], raising the question of whether distinct macrophage types associate with distinct prognostic behaviors. Dissecting TAM heterogeneity using single-cell approaches, including, for example, single-cell RNA sequencing, could help identify more informative prognostic biomarkers and possibly yield novel and more specific therapeutic targets. 

## 3. Macrophages in Metastases

The majority of studies probing the clinical relevance of immune cells in colorectal cancer primarily [6] and in metastatic CRC [51,52] have focused on adaptive immune cells. However, macrophages are gaining attention, although inconsistencies among studies performed on preclinical models and human studies are evident. Mostly in preclinical studies, macrophages have been shown to pave the way to tissue invasion and intravasation and to provide a nurturing microenvironment for metastasis, serving as a component of the cancer cell niche at distant sites [53]. TAMs release a plethora of extracellular matrix (ECM) remodeling factors (plasminogen activation system, matrix metalloproteinases, and kallikrein-related peptidases), thereby affecting the composition, structure, and elasticity of the ECM and the availability of growth factors, creating conduits for the migration of tumor cells [54]. During metastasis, invasive tumor cells need to survive and grow in a hostile environment by establishing a premetastatic niche. Metastasis-associated macrophages (MAMs), in murine breast and CRC models, have been shown to increase the extravasation of tumor cells and help in their survival by secreting growth factors and concomitantly inhibiting cytotoxic T cells [42]. In mice, metastatic cells recruit Ly6C+ monocytes (CD14hiCD16− monocytes in humans) through the classical CCL2–CCR2 axis. Instead, once in tissues, the CCR1–CCL3 autocrine signaling pathway was shown to be critical for differentiation into MAMs [55]. In lung metastasis, MAMs, characterized by the surface markers CD11b, VEGF receptor 1 (VEGFR1), CXCR3, and CCR2, support the survival of metastatic cells through a vascular cell adhesion protein 1 (VCAM1)-dependent and AKT-dependent mechanism [56,57]. On the other hand, metastatic cells promote the retention of MAMs in the metastatic niche, thereby further aiding tumor cell survival [55]. A similar mechanism takes place in bone metastasis; metastatic cells activate osteoclasts, the resident macrophage population of the bone, to free the growth factors contained in the bone [58]. 

### 3.1. Immune Landscape of Human CLM

Colorectal cancer is a major cause of mortality worldwide [59]. Most of colorectal cancer patients develop CLM either at time of the initial diagnosis or later after the resection of the primary tumor [60], mostly due to anatomical factors. The liver, in fact, filters the majority of the intestinal drainage which enters it through the hepatic portal venous system [61]. Nowadays, for CLM patients, hepatic resection combined with systemic chemo-immunotherapy has been associated with 5- and 10-year overall survival rates up to 50% and 35%, respectively, and, therefore, has the potential to be curative [61]. Traditional prognostic factors have been used to stratify the clinical outcome of CLM patients after hepatic resection, albeit with heterogeneous results [62]. These features are informative tools to predict the prognosis of CLM patients; however, the wide spectrum of clinical presentations and varying degrees of responsiveness to therapy of metastatic colorectal patients require the development of new and more reliable prognostic markers. In this regard, the immune landscape could provide a promising tool to help in the stratification of clinical outcome.

The liver is an organ with a central role in host defense and systemic inflammation. Because of its anatomical location and structure and its physiological function, it is continuously exposed to food antigens, pathogens, and endogenous compounds. In homeostasis, immune responses are suppressed through several mechanisms, ensuring an immunologically tolerant microenvironment. Upon inflammatory stimuli, tissue-resident macrophages and hepatic stellate cells release high amounts of pro-inflammatory cytokines and chemokines, resulting in the recruitment and accumulation of neutrophils, monocytes, NK cells, and natural killer T (NKT) cells. There are increasing clinical data evidencing an impact of leucocyte populations on the clinical outcome of patients with colorectal liver metastases [6,51,63,64,65]. The impact of immune infiltration in primary colorectal tumors has been investigated thoroughly, revealing a role for infiltrating T cells in predicting prolonged patient survival [6,8,51,66]. This solid evidence strongly supports the introduction in clinical routine of the evaluation of the immune infiltrate for a better classification of colorectal cancer patients. However, the immune landscape within colorectal liver metastases and the impact of immune cells on clinical outcome in metastatic patients have been, until recently, far less explored.

The predictive potential of adaptive immune cells has been investigated also in CLM, evidencing CD8 and CD4 T cell densities as powerful prognostic tools [51,67]. Moreover, in a recent work, Donadon et al. assessed the clinical relevance of T cells and NK cells in the microenvironment of CLM patients, which were found to associate favorably to overall survival [68].

In the context of colorectal liver metastases, a major issue is represented by the wide array of clinical presentation of the patients, which is mirrored by the heterogeneity in the response to treatments [62]. The issue of heterogeneity in CLM has recently been extensively addressed [51,52]. These comprehensive retrospective studies have brought to light the extreme diversity of metastases between patient, and within the same patient. Indeed, metastases of the same patient presented with markedly different T cell infiltration, ranging from highly infiltrated (“hot” tumors) to scarcely infiltrated (“cold” tumors), and variable degrees of response to pre-surgical therapy. However, in these studies the impact of myeloid cells, which could also play a role both in clinical outcome and in responsiveness to therapy, was not taken into account. 

Collectively, these studies highlight the importance of assessing the immune microenvironment as a biomarker of patient prognosis and response to therapy, bearing in mind the inter- and intra-metastatic immune and genetic heterogeneity and the possible impact of neoadjuvant regimens.

### 3.2. Focus on Macrophages in Human CLM

Hepatic-resident macrophages, Kupffer cells (KCs), have long been studied for the numerous crucial functions they perform to maintain homeostasis in the liver and in the whole body [69]. Kupffer cells are endowed with a high phagocytic ability, required for scavenging pathogens and dangerous endogenous compounds, and for the recycling of iron from senescent red blood cells [15]. However, in recent years, it has become increasingly clear that hepatic macrophages are a heterogeneous population composed mainly of Kupffer cells, self-renewing and long-lived, and monocyte-derived macrophages, mainly residing in the portal triad, involved in cholesterol metabolism and iron recycling [69]. Whereas KCs generally display a tolerogenic and immunosuppressive phenotype, monocyte-derived macrophages are characterized by a pro-inflammatory gene expression profile [70]. Deciphering macrophage heterogeneity could have interesting implications for the development of new therapeutic strategies. Indeed, immune monitoring in metastatic colorectal patients identified circulating CCR2^+^ monocytes as prognostic biomarkers associated with worse patient prognosis. Accordingly, inhibition of CCR2 resulted in increased response to chemotherapy and improved overall survival in a preclinical model [71]. Along the same lines, CCL5 was shown to recruit and skew macrophages towards a pro-tumor phenotype; blocking the corresponding receptor CCR5 induced tumor responses in a phase I clinical trial in advanced colorectal patients [72]. However, from the available data in CLM patients, the role of macrophages is still debated, possibly due to the heterogeneous composition of hepatic macrophages. In fact, in a retrospective study, a high CD68^+^ TAM density was associated with longer patient disease-free survival (DFS) [49]. Furthermore, chemotherapy treatment was shown to increase the levels of circulating intermediate monocytes (CD14^++^/CD16^+^), and this parameter was predictive of response to chemotherapy but not to anti-VEGF treatment [73]. 

## 4. Concluding Remarks

Despite the wealth of studies addressing the role and clinical relevance of the immune microenvironment in primary colorectal cancer, far less is known concerning its role in liver metastases. Yet, surgical resection of CLM has the potential to be curative, thus attention is given to the identification of new prognostic factors. Recent studies have shown that high densities of T cells and NK cells are associated with a favorable clinical outcome, while the role of macrophages is still debated. TAMs have been shown to promote tumor growth and metastatization, help establish a pre-metastatic niche further promoting tumor growth through the secretion of growth factors, and modulate the efficacy of conventional and unconventional anticancer strategies. However, contradictory associations with prognosis have been reported. This could be due to the fact that hepatic macrophages are a heterogeneous population, composed mainly of resident Kupffer cells and monocyte-derived macrophages, with different phenotypic profiles. Until now, the prognostic relevance of macrophages has largely relied on the use of the pan-macrophage marker CD68, which, however, does not take into account the heterogeneity of TAMs. Therefore, a more refined approach to quantify TAM response in CLM is still greatly needed. In fact, deciphering TAM diversity in CLM, for example using single-cell based approaches, could help design more individualized therapeutic approaches. 

## Figures and Tables

**Table 1:** Tumor-associated macrophage infiltration as a predictor of patient outcomes in colorectal cancer.

Tumor Stage	Cohort	Marker	Prognostic Value	Reference
Colorectal cancer (stage II–III)	131	CD68/VEGF	Positively correlated with OS (*p* = 0.01)	Khorana et al. (2003) [44]
Colorectal cancer (ND)	60	CD68	Positively correlated with OS (*p* < 0.01)	Tan et al. (2005) [45]
Colorectal cancer (stage II–III)	310	CD68	None	Bacman et al. (2007) [46]
Colorectal cancer (ND)	446	CD68	Positively correlated with CSS (*p* = 0.007)	Forssell et al. (2007) [13]
Colorectal cancer (stage III)	98	CD68	Positively correlated with OS (*p* = 0.041)	Zhou et al. (2010) [47]
Colorectal cancer (ND)	485	NOS2 (M1-like)	Positively correlated with CSS (*p* = 0.0003)	Edin et al. (2012) [14]
		CD163 (M2-like)	Positively correlated with CSS (*p* < 0.0001)	
Colorectal cancer (stage II–III–IV)	159	CD68	Positively correlated with DSS (*p* = 0.02 univariate analysis)	Algars et al. (2012) [48]
		CLEVER-1/stabilin-1 ^§^	Positively correlated with DSS (*p* = 0.04 univariate analysis)	
Colorectal cancer (stage III)	208	CD68	Positively correlated with DFS (*p* = 0.005) ^ç^	Malesci et al. (2017) [10]
Colorectal cancer liver metastases (CLM)	158	CD68	Positively correlated with DFS (*p* = 0.023)	Cavnar et al. (2018) [49]
Colorectal cancer (stage I–II–III–IV)	419	CD68	Positively correlated with OS (*p* = 0.003)	Li et al. (2018) [50]

OS, overall survival; CSS, cancer-specific survival; DSS, disease-specific survival; DFS, disease-free survival; ND, not defined; ^§^ CLEVER-1/stabilin-1 was used as a marker of regulatory/suppressive macrophages; ^ç^ positive correlation occurred only in patients treated with Fluorouracil (5-FU) adjuvant therapy.
